# Nursing staff perspectives of continuous remote vital signs monitoring on surgical wards: Theory elicitation for a realist evaluation

**DOI:** 10.1111/jep.13678

**Published:** 2022-04-03

**Authors:** Candice Downey, Julia Brown, David Jayne, Rebecca Randell

**Affiliations:** ^1^ Leeds Institute of Medicine at St. James's, St. James's University Hospital University of Leeds Leeds UK; ^2^ Leeds Institute of Clinical Trials Research University of Leeds Leeds UK; ^3^ Faculty of Health Studies University of Bradford Bradford UK; ^4^ Wolfson Centre for Applied Health Research Bradford UK

**Keywords:** nursing, realist evaluation, remote monitoring, vital signs

## Abstract

**Rationale, Aims and Objectives:**

Continuous remote monitoring (CRM) provides a novel solution to the challenges of monitoring patients' vital signs in hospital, but the results of quantitative studies have been mixed. Acceptance by staff is a crucial determinant of the success of healthcare technologies and may explain these discrepancies. Drawing on the approach of realist evaluation, this paper aims to identify theories about how, why and in what conditions nursing staff perceptions vary regarding the CRM of patients' vital signs.

**Methods:**

Multiple methods were used to elicit theories about factors likely to facilitate or impede the successful implementation of continuous remote vital signs monitoring. This included a literature review, consultation with patients and observational work conducted during a randomized controlled trial (RCT) of CRM. In addition, a priori theories developed through informal interactions with patients and ward staff during the day‐to‐day set‐up of the trial were included.

**Results:**

The findings suggest that the perceptions of nursing staff regarding remote monitoring can be influenced by the type of patients under their care and their previous experience of telemetry. Factors which may undermine the engagement of staff are perceived staff burden, which can be dependent on contextual factors such as staffing levels, time of day and senior staff attitudes. Staff attitudes are also likely to be influenced by patient perspectives and the utility of the devices associated with remote monitoring. The successful implementation of CRM may be dependent on staff training, research staff input and hospital culture.

**Conclusions:**

Theories regarding nursing staff engagement with remote monitoring are numerous, varied and contradictory. The theories elicited in this initial phase will be refined during interviews with the nursing staff involved with the RCT.

**Abbreviations**
CMO: context‐mechanism‐outcome
CRM: continuous remote monitoring
NEWS: National Early Warning Score
RCT: randomized controlled trial
REC: Research Ethics Committee

## 1 INTRODUCTION

The successful implementation of new healthcare technologies into routine clinical practice is predicated on engaging both staff and patients.^1^ It is crucial to assess the experiences of the people using the technology to identify contextual factors that support or constrain optimal utilization, which could influence the effectiveness of the device.^2^

The remote monitoring of patients' vital signs is an area of increasing interest due to the innate limitations of manual vital signs monitoring in hospital.^3^ The SensiumVitals® remote monitoring system consists of a patch worn on the patient's chest and continuously measures heart rate, respiratory rate and temperature. These data are transmitted wirelessly to a mobile device which alerts the nurse if the vital signs stray outside of normal parameters, potentially allowing earlier detection and treatment of patient deterioration in hospital. This technology has been evaluated in two feasibility studies in the surgical population.^4^, ^5^ The TRaCINg feasibility randomized controlled trial (RCT) compared usual intermittent vital signs monitoring, in the form of the National Early Warning Score (NEWS), and continuous remote monitoring (CRM) in addition to NEWS.

The small number of quantitative studies in the field of continuous monitoring have shown mixed results.^6^ The success of these technologies is context‐dependent and reliant on both patient and practitioner engaging effectively with the technology. We have previously studied the perceptions of patients regarding continuous vital signs monitoring in hospital^7^; in this study, we undertook semi‐structured interviews with surgical inpatients as part of a study testing a remote continuous monitoring device and analysed the results using thematic analysis.

Realist methods were chosen to determine the perceptions of staff members, given that acceptance by staff may be the single most important determinant of the success of healthcare technologies at a local level.^1^ Realist evaluation is a theory‐driven approach to the evaluation of complex interventions in healthcare.^8^ It is based on the idea that interventions (such as a new monitoring system) offer resources to people, but it is how people choose to respond to the resources that determine their impact, and such choices are highly dependent on context.^9^ Realist evaluation aims to explain why the intervention works in some circumstances, but not in others. It involves eliciting stakeholders' theories and then gathering empirical evidence to test and refine those theories. In realist evaluation, the term ‘theory’ refers to participants' ideas and thoughts about how an intervention works, based on their everyday experience. This type of ‘informal theory’ is always at work in improvement work, although practitioners are often not aware of it or do not make it explicit.^10^ Staff are the users of the monitoring system from a realist perspective, and we were interested in their response to the system as this will determine the impact of the intervention on patients.

This paper presents the theory elicitation phase of the realist evaluation that was undertaken alongside the TRaCINg feasibility RCT. The theory elicitation phase aimed to identify stakeholders' theories concerning how, why and in what conditions continuous remote vital signs monitoring is optimally used on the surgical wards of a large teaching hospital. Elucidation of these contextual factors and their effects will inform potential wider implementations of this technology and may reveal strategies to support staff in the future.

## 2 METHODS

The first phase of a realist evaluation is that of theory elicitation.^11^ Realist theories are presented in context‐mechanism‐outcome (CMO) configurations, with the mechanisms divided into resources and responses. In the case of CRM, the technology provides a fixed resource; it is the response to the resource that determines if the desired outcomes are achieved. This response is determined by the context in which the resource is implemented; for instance, the clinical area itself, or the experience levels of the staff employed there. As an example, in the *context* of engaged senior colleagues, staff nurses may *respond* by carrying the devices and acknowledging alerts appropriately, leading to recognition of the deteriorating patient (the desired *outcome*).

Multiple methods were used to elicit theories about factors likely to facilitate or impede the successful implementation of continuous remote vital signs monitoring. This included a literature review, consultation with patients and observational work conducted during the TRaCINg study. In addition, a priori theories developed by CD through informal interactions with patients and ward staff during the day‐to‐day set‐up of the study were included.

### 2.1 Literature review

MEDLINE®, MEDLINE® In‐Process, EMBASE, CINAHL and The Cochrane Library were searched for articles published from the dates of inception of the databases (the earliest being 1947) to October 2017. The search strategy is detailed in Supplementary Materials, including the criteria for the selection of studies and methods of data extraction and synthesis.

In brief, the selection and appraisal of identified papers were based on relevance to the review question, as is the case in the theory elicitation phase of a realist review.^12^ Papers were included if they contained theories about staff perceptions regarding CRM of patients' vital signs. These included empirical studies, theoretical literature, review articles and grey literature. Quality appraisal of the selected papers was not undertaken as the purpose was solely to identify potential theories to be refined in later stages of the research, rather than evaluate the truth of the theories at this stage. Theories and theory fragments were extracted from the literature and then grouped together and refined as the review progressed. Conflicting theories were also included, with care being taken to note the context in which these contradictory ideas were founded.

### 2.2 Patient consultation

Patients' ideas about nursing perceptions of CRM were gleaned from face‐to‐face interviews at the hospital beside,^7^ informal interactions during the day‐to‐day management of the TRaCINg study and two patient focus groups conducted as part of the Patient and Public Involvement work ahead of the feasibility trial. The full methodology of the patient interviews has been published elsewhere.^7^ The topic guide for the focus groups was developed for this study and is provided as Supplementary Material. Data from the transcripts of the interviews and focus groups were coded to identify themes in the participants' responses. These codes were then refined to identify patient theories, which were added to those identified in the literature review.

### 2.3 Nonparticipant observation

During the TRaCINg study, CD dedicated approximately 20 h to observation of the ward staff during vital signs monitoring. During daily visits to the wards, field notes were taken to document staffing levels and the proportion of senior nursing staff on shift, alongside informal comments from ward staff and observations of interactions between and within staff members and patients, and with the technology itself. These field notes were reviewed after the end of the TRaCINg study and coded to identify common themes, which were further refined to draw out new theories concerning the perceptions of nursing staff with regard to the CRM devices. These theories were added to those identified through the literature review and patient consultation alongside a priori theories developed by CD through informal interactions with patients and ward staff during the day‐to‐day set‐up of the study.

## 3 RESULTS

### 3.1 Literature review

The search retrieved over 1000 references. After the selection process, a total of 84 sources were identified. Three papers were systematic reviews of studies of continuous vital signs monitoring; one article was a nonsystematic review. There were 25 individual studies of CRM, including both quantitative and qualitative data. These were evaluated together with 16 editorials and 39 websites. There was considerable repetition of theories across the sources identified. These theories largely fell within three larger themes: nursing perceptions of CRM, the development of CRM technologies and the implementation of CRM technologies.

#### 3.1.1 Theories regarding nursing perceptions of CRM

Five studies specifically reported nursing perceptions of CRM systems^13^, ^14^, ^15^, ^16^, ^17^ and all identified similar themes. In general, nursing staff could see the potential for continuous monitoring to enhance patient safety. Nurses perceived that greater ‘availability and accessibility’ of vital signs information would support their decision‐making and provide reassurance to patients.^15^ Context did appear to have a role in determining the perceptions of nursing staff. Jeskey et al.^16^ found that nurses with prior telemetry experience were more likely to perceive the monitoring device as beneficial and more clearly understood the device. It was also suggested that the devices were perceived to be more beneficial by night staff rather than during day shifts, potentially due to reduced staffing levels and more frequent monitoring of high‐acuity patients ‘in the immediate postsurgical period’.^16^ An alternative theory was that in the context of night shifts, the increase in patient: staff ratios may lead to the devices being perceived as an addition burden (response), causing failure to engage (outcome).

Two papers reported that nurses were worried that visibility of information and alarms would cause patient anxiety, leading to increased time spent to reassure them.^15^, ^18^ Both of these studies were conducted on respiratory wards, which may have high‐acuity and therefore high‐anxiety patients. However, the visibility of information on CRM devices was also considered to provide opportunities for increased engagement of patients in their own care.

Prgomet et al. reported concerns from both doctors and nurses about over‐reliance on CRM leading to decreased bedside interactions.^15^ A conflicting yet recurring theme across the literature was that of staff burden. Van Loon et al. highlighted the fact that CRM devices typically collect large amounts of information, which has the potential to overwhelm users and dilute important indicators of deterioration.^18^ Other studies reported concerns that CRM overburdens busy ward staff or takes nursing staff away from other tasks.^19^ This was particularly evident during day shifts, when staff are typically busy with a wider variety of duties than during night‐time hours.^16^ The underlying theory appears to be that In the context of a busy ward environment, the nurses will be too busy for an extra task and will fail to engage with the devices (their response), leading to clinical deterioration going unrecognized (the outcome).

Eight studies reported concerns about alert burden. These studies shared a common context of high acuity patent populations and higher patient: nurse ratios. Banks et al. found such a problem with nuisance alarms that monitoring had to be abandoned because of nursing complacency towards the alarms.^17^ Alarm fatigue and data inaccuracy were also reported by Jeskey et al., who found that excessive false‐positive alerts interrupted nurses and distracted them from other responsibilities.^16^ There was also concern that doctors might become overburdened and desensitized to calls.^15^ This suggests that in the context of very sensitive devices, there will be a high number of false alerts, leading to alert fatigue, desensitization and failure to respond to alerts (the response), with the outcome of unrecognized deterioration.

#### 3.1.2 Theories regarding development of CRM technologies

Three articles commented on the limitations of current CRM devices, outside of concerns about false alerts. Patient comfort was a priority^15^, ^18^; the underlying theory appears to be that in the context of patients finding the devices uncomfortable, or feeling anxious wearing them, the nursing staff may consider the devices to offer more harm than good, with the outcome that they fail to engage with the CRM technology.

It is also suggested that nursing staff should also feel comfortable with the devices^20^ to avoid losing confidence in the technology as a whole. In the context of nurses lacking confidence when using new technology, their response will be to fail to engage with the devices leading to the outcome of unrecognized clinical deterioration.

Other theories suggested that merely notifying caregivers of abnormal readings are inadequate and that usability of devices would be improved by incorporating a suggested action in response to notifications,^18^ especially where devices collect a large amount of data for interpretation. In the context of the devices gathering large amounts of information, the nursing staff may feel overwhelmed (response) leading to a lack of confidence when interpreting and acting on alerts (outcome). In addition, in the context of the devices not suggesting an action after an alert, the nursing staff may not know how to respond to the alert and may subsequently fail to act on notifications. Basing suggested actions on local policy could enhance perception of CRM as integrated into the usual care pathway.^20^ If the CRM system is not incorporated into local protocols and policies (context), nursing staff may be ambivalent towards the technology (response) and fail to engage (outcome).

Another potential way to improve integration is to remove notification devices from individuals and instead promote a ward‐based responsibility for CRM, by incorporating big screens at the nurses' station.^21^, ^22^ In the context of wards being divided into sections, each of which is the responsibility of a single nurse, the nurses may perceive the device as an individual burden, rather than as a collective responsibility, leading to disengagement from the system and decreased responsiveness to alerts. Allowing ward‐based responsibility could help overcome another limitation of individual nurse responders: in the context of nursing staff only seeing the benefit of the CRM system on a patient‐by‐patient basis, or only in patients who have deteriorated, they may underestimate the global impact of the devices (response) with the outcome that their engagement with the CRM devices is impaired.

#### 3.1.3 Theories regarding implementation of CRM technologies

A number of theories emerged regarding nursing perceptions of the optimal strategy for implementation of CRM technologies. In the literature, these theories were most often found in the nonsystematic review by Taenzer et al. First and foremost was the theory of optimizing the intervention as much as possible before implementation to avoid examples of early technology failure which might lead to mistrust from end users.^20^ In the context of previous failed iterations of the CRM devices, the staff may not trust the new technology (response) and may fail to engage with it (outcome). If nursing staff have experience where vital signs monitoring failed to detect patient deterioration, or if their detection of abnormal vital signs failed to elicit the appropriate clinical response (context), they may consider CRM monitoring to be superfluous (response) and fail to engage (outcome).

Another suggested tactic to improve engagement of early adopters was incentivizing staff to use the devices appropriately^23^; suggested incentives ranged from updating staff about recent patient success stories, ranking wards against each other or providing ‘gifts' to highly engaged teams. In the context of staff not having incentives to respond to alerts, they may not be motivated to engage with the devices (response) with the outcome that they do not respond to alerts.

Other theories concerned the context of initial implementation. One broad idea was the need to ensure that innovation is supported in the local hospital culture.^20^ If research and innovation are not supported in the local hospital culture (context), nursing staff may be intolerant of new devices (response) and fail to engage with the CRM technology (outcome).

In the case of CRM, pilot ward/patient selection emerged as a recurring theme. Jeskey et al. found a more positive perception of CRM in nurses looking after higher‐acuity patients, such as those just back from surgery.^16^ A conflicting theory emerged in that high‐acuity wards often have a high turnover of staff, which may cause difficulties when trying to implement a new intervention which requires initial training and sustained engagement. In the context of high acuity wards, the staff are extremely busy with clinical duties and may be unable to manage the extra burden of remote monitoring, leading to a failure to engage with the technology. In this context, vital signs monitoring may be delegated to healthcare assistants, leading to qualified nurses perceiving vital signs as not part of their work (response) and failing to engage with monitoring technology (outcome).

Embedding new technology within existing local processes was another recurring theme. Nursing staff is potentially more likely to successfully integrate CRM into their working practices if it is incorporated into local monitoring protocols alongside explicit escalation guidance.^18^, ^20^ To this end, it might be helpful to extend staff training in the new technology to non‐ward‐based staff such as doctors and outreach teams.^15^ If vital signs monitoring is considered to be an exclusively nursing task (context), the nurses will not be empowered to use the CRM alerts when escalating a patient to doctors (response), and will therefore no longer consider the monitoring systems to be worthwhile within their care protocols (outcome). Incorporating CRM alongside traditional observations could increase perceptions of its utility, encourage nursing staff engagement and incite wider institutional acceptance.

### 3.2 Patient consultation

The full analysis of the patient interviews and focus groups has been reported previously.^7^ A predominant theme emerging from the patient interviews regarding nursing responses was concerns about workload. Nursing staff was described as ‘too busy’ and ‘on their feet all the time’. Patients expressed that they saw CRM as having value for nursing staff in terms of freeing up nurses' time for other tasks. One patient said, ‘[The nurses] can use this gadget – they don't have to do as many visits… to your bedside’. Another echoed this theory: ‘The nurses could get on with other things… so it saves time for them’. A conflicting theory emerged from the focus groups. Patients were concerned that the extra monitoring would increase workload. This was mentioned in combination with the theory of false alerts causing interruptions and distractions from essential tasks: ‘I'd think [the nurse] would have enough to do, without pandering to me’.

### 3.3 Nonparticipant observation

This was a particularly rich source of theories which incorporated informal, ‘throwaway’ comments from ward staff and close observation of interactions between and within staff members and patients. One of the most striking observations was the impact of the attitudes of senior nursing staff on ward engagement with CRM. In wards where the Nurse in Charge was ambivalent about the technology, staff engagement required substantially more researcher input when compared to wards where the senior nurse was enthusiastic about the devices and their potential. Senior staff engagement may be a crucial component when considering how to implement new technology at ward level. If senior nurses are dismissive of the remote monitoring technology (context), junior staff perceive the devices as unnecessary (response), leading to a failure to engage with the system (outcome).

Another important observation emerged when new staff members started work on the wards and highlighted issues regarding staff training. Before commencement of the TRaCINg study, nursing staff was trained in a single, hour‐long drop‐in session, with sessions available throughout a single week. There was no provision for formal training for staff who started work after the training period. In addition, there were a number of staff members who requested ‘refresher’ training during the TRaCINg study. In the context of training being provided over a single session, staff may lack confidence (response) and fail to engage with the technology (outcome). This highlighted the importance of regular training opportunities to keep up with the high level of staff turnover and the need for retraining of current staff. In the context of high staff turnover on busy wards, new staff may not be trained of confident to use the CRM monitoring (response) and clinical deterioration could go unrecognized (outcome).

A similar theme emerged when technical problems occurred with the CRM devices. The absence of on‐site technical support for minor issues led to loss of confidence and rapid disengagement by one affected staff member, as evidenced by her reluctance to carry the device during the rest of the trial. In the context of a lack of on‐site technical support, technical malfunctions could not be rectified immediately, leading to a loss of confidence in the technology (response) and failure to engage (outcome).

An unanticipated theory emerged from nursing staff working with older patients. Nurses were reluctant to use the CRM devices within view of their patients because they resembled mobile phones; nursing staff perceived that their patients would assume they were undertaking personal tasks rather than clinical work. In addition, staff would turn down the volume of the alerts so patients could not hear them, in case patients mistook the alarms for personal messages. This led to a delay in responding to some notifications. In the context of an older inpatient population, and devices which appear similar to personal mobile phones, nursing staff may feel self‐conscious using the system in front of patients (response) and refuse to carry the devices, or check notifications on the ward (outcome). This may have implications for future device development.

### 3.4 A priori theories

Theories were developed by CD through informal interactions with patients and ward staff during the day‐to‐day set‐up of the study. These were broad speculative concepts regarding nursing staff's perception of CRM and vital signs monitoring as a whole. They included a number of conflicting theories. One such theory concerns the value of vital signs to nursing staff. Some papers have suggested nurses consider vital signs monitoring to be inadequate in the detection of patient deterioration, or not part of the work of a staff nurse, given that most observation rounds are delegated to healthcare assistants. This raised the question of whether CRM would address these concerns by provision of more data, or simply provide more perceived unnecessary information. In addition, if nursing staff lack confidence in the efferent arm of the deteriorating patient pathway, it would be difficult to perceive additional monitoring as providing any downstream patient benefit.

A conflicting theory is that nursing staff perceive traditional vital signs monitoring to be sufficient to detect patient deterioration. This may be reinforced by the fact that national guidance currently dictates frequency of manual vital signs observations. In this case, CRM is likely to have little perceived benefit. Instead, it may be perceived as a threat to autonomy when deciding whether to escalate unwell patients. In the context of national guidance (the NEWS protocol) mandating the frequency of manual observations, nursing staff may respond by thinking that current observation intervals are sufficient (response), and fail to perceive the benefit of continuous monitoring over normal care, leading to a lack of engagement with the devices (outcome).

Theories were also developed regarding implementation of the CRM technology. In the TRaCINg study, the research team was removed from ward‐level monitoring but provided weekday technical assistance by undertaking the application, replacement and removal of the CRM devices when necessary. One theory was that in the context of a research study, by removing these tasks from the ward staff, they might perceive CRM as outside of their responsibility (response) and fail to engage with the technology (outcome). It was anticipated that this would potentially be more evident on high‐acuity wards where the nursing staff may feel they are unable to manage the extra burden of CRM. This would be compounded at weekends, when the research team is absent, and if devices were perceived to be difficult to use. If the devices are difficult to use (context), the nursing staff may not be confident in using the technology (response) and fail to engage with it (outcome).

Table 1 summarizes the elicited theories at the end of the theory elicitation phase as CMO configurations.

**Table 1:** A summary of the theories elicited, expressed as context‐mechanism‐outcome configurations

Source	Context	Mechanism		Outcome
		Resource	Response	
Patient interviews	Nurses too busy for an extra task	‐	Nurses fail to engage with devices	Clinical deterioration goes unrecognized
Patients and literature	CRM devices are programmed to be very sensitive to patient deterioration	A high number of false alerts^19^, ^20^, ^21^	Alert fatigue, desensitization and failure to respond	Clinical deterioration goes unrecognized by staff
Literature	Nurses not confident with technology	Devices require some technical capabilities	Nurses fail to engage with devices	Clinical deterioration goes unrecognized
	Vital signs monitoring is considered to be exclusively a nursing task	Training in CRM is specific for nursing staff^15^	Nurses unable to use remote monitoring vital signs when triggering escalation protocols	Nursing staff don't consider remote monitoring to be worthwhile
	‐	There is a large amount of information gathered by the remote monitoring devices^18^	Nursing staff feel overwhelmed by information compared to NEWS	Nursing staff lack confidence when interpreting and acting on notifications
	‐	There is no suggested action for notifications^18^	Nursing staff do not know how to respond to notifications	Nursing staff failed to act on notifications
	Patients find devices uncomfortable, or feel anxious being continuously monitored^15^, ^18^	‐	Nursing staff consider devices offer more harm than good	Failure to engage with remote monitoring technology
	Nurses are engaged in other tasks^15^	Remote monitoring notifications take nursing staff away from other tasks^19^	Nursing staff get frustrated by interruptions. Nurses prioritize other tasks over responding to alerts	Usual tasks take longer due to interruptions. Frustrated nursing staff fails to engage with the devices. Clinical deterioration goes unrecognized
	There is a high rate of staff turnover on high‐acuity wards^20^	New staff are not aware of the remote monitoring devices	New staff do not use remote monitoring as per protocol	Clinical deterioration goes unrecognized
	Wards are divided into sections, each of which is the responsibility of a single staff nurse	Nurses are solely responsible for the remote monitoring receiving device for their section^21^, ^22^	Nurses perceive device as an individual burden	Decreased responsiveness to alerts
	Nursing staff only see benefit/burden on a patient‐by‐patient basis^23^		Nursing staff failed to appreciate global impact of device	Failure to engage with remote monitoring technology
	Nursing staff only perceive benefit in patients who have deteriorated^24^	Devices are silent in patients with normal vital signs	Nursing staff failed to appreciate global impact of device	Nursing staff ignore ‘low‐risk’ patients
	Nurses are not incentivized to respond to alerts^23^		Nursing staff are not motivated to engage with devices	Nursing staff do not respond to alerts
	Continuous monitoring is not included in local policy documents^20^	Nursing staff perceive NEWS as sufficient to detect deterioration	Nursing staff ambivalent about continuous monitoring	Failure to engage with remote monitoring technology
	Research and innovation is not supported in the local hospital culture^20^	‐	Nursing staff are intolerant of novel devices	Failure to engage with remote monitoring technology
	Previous iterations of continuous monitoring have been poorly implemented^20^	Nursing staff have seen examples of technology failure^25^	Nursing staff do not trust the new technology	Failure to engage with remote monitoring technology
Observations	Training provided over a single session	Staff insufficiently trained	‐	Nursing staff not confident with technology
	Nursing staff workload is higher in daytime hours, but nurse:patient ratios are lower at night	‐	Nursing staff perceive continuous monitoring as a burden on over‐stretched staff throughout the 24‐h day	Nursing staff failed to exchange the devices at handover periods at the end of a shift. Failure to engage with remote monitoring technology
	There was no on‐site technical support available	Technical malfunctions could not be rectified immediately^20^	‐	Loss of confidence in the technology and failure to engage
	Nursing staff frequently attend to older patients	Remote monitoring devices look like mobile phones	Nursing staff are afraid that patients will assume the devices are their personal phones	Nursing staff refuse to carry the devices or check notifications on the ward
	Senior nurses dismissive of remote monitoring technology	‐	Staff nurses perceive remote monitoring as unnecessary	Failure to engage with remote monitoring technology
A priori theories	Diminished researcher presence at weekends	‐	Staff nurses forget about study	Failure to collect monitoring devices
	Remote monitoring implemented on a ward with high‐acuity patients	Staff are extremely busy with clinical duties	Staff unable to manage the extra burden of remote monitoring	Failure to engage with remote monitoring technology
	Nursing staff have experience of vital signs failing to detect deterioration	‐	Nursing staff consider vital signs to be inadequate in detecting deterioration Nursing staff cannot perceive any downstream patient benefit from improving vital signs monitoring	Failure to engage with remote monitoring technology
	Context in which nursing staff perceive CRM as a replacement for EWS?	‐	Remote monitoring is perceived as a potential replacement for manual observations. Nurses perceive remote monitoring as a threat to autonomy	Nurses avoid using remote monitoring in their patients
	Nursing staff are busy undertaking skilled tasks	Healthcare assistants are in charge of collecting vital signs	Staff nurses perceive vital signs as not part of their work	Failure to engage with remote monitoring technology
	Nursing staff have bad experiences of the efferent arm of the deteriorating patient pathway	Escalation protocols	Nursing staff does not have confidence in the efferent arm of the deteriorating patient pathway. Nursing staff cannot perceive any downstream patient benefit from improving vital signs monitoring	Failure to engage with remote monitoring technology
	National guidance dictates frequency of manual observations	‐	Nursing staff feel that current observation intervals are sufficient	Nursing staff fail to perceive the benefit of continuous monitoring over normal care
	Continuous monitoring is implemented as part of a research study	Research staff are responsible for patching patients	Staff nurses perceive remote monitoring as not part of their work	Failure to engage with remote monitoring technology
	‐	The devices are difficult to use	Nursing staff are not confident using the technology	Failure to engage with remote monitoring technology

Abbreviations: CRM, continuous remote monitoring; NEWS, National Early Warning Score.

## 4 DISCUSSION

This paper presents the results of the theory elicitation phase of a realist evaluation, conducted alongside a feasibility RCT of continuous remote vital signs monitoring versus intermittent manual observations alone. This realist evaluation is the first of its kind to identify theories about how, why and in what conditions nursing staff perceptions vary regarding the CRM of patients' vital signs.

The theory elicitation phase has provided a number of theories to be refined in the next phase of the study. We have focused on the contextual factors that affect engagement with CRM technology, as these are where we can implement change. These can be subdivided based on factors described in Davis's technology acceptance model (TAM), which is the most widely applied model of users' acceptance and usage of technology.^26^ The model consists of perceived usefulness, perceived ease of use and attitude towards the technology,^27^ which determine the clinical and nonclinical efficacy of CRM systems. Realist evaluation often draws on existing theory such as Davis' TAM, and the theories elicited in this study can be readily subsumed by this model. In terms of perceived usefulness, the results suggest that nursing staff can see the potential of CRM to be useful in enhancing patient safety, although this understanding can be influenced by contextual factors such as the type of patients under their care and their previous experience of telemetry.

Factors determining ease of use include staff comfort and staff burden. The theories suggest that prioritizing the comfort of the nursing staff when developing CRM technologies will enhance staff engagement with the devices. Key contextual factors which require discussion centre around the appearance of the devices, the use of prompts, communal ward‐based screens and integration into local care pathways. Staff burden can be dependent on contextual factors such as staffing levels and time of day. The successful implementation of CRM may be dependent on the context of staff training, research staff input and hospital culture.

Staff attitudes towards the technology are likely to be influenced by patient perspectives and senior staff attitudes. In different contexts, patients may be reassured or made anxious by the extra monitoring, depending on its impact on the number of bedside interactions between staff and patient. Senior staff engagement may be a crucial component when considering how to implement new technology at ward level.

A strength of this study is the comprehensive and multiple methods use to elicit theories, including a literature review, patient consultation and real‐time observations of nursing practice through daily wards visits as part of the TRaCINg study. This allowed a wide range of theories to be elicited, including contradictory ideas. However, these theories remain to be tested. The next phase of the study will comprise semi‐structured interviews with the nursing staff involved in the TRaCINg study. The initial theories, developed in Phase 1, will be compared and contrasted with the nursing staff perspectives gathered in Phase 2 and synthesized to offer explanations as to how nursing staff perceive and subsequently implement the CRM system and the contextual factors that influence this. The refined theories can then be prioritized for testing in a definitive evaluation of CRM, to explain the causal mechanisms which produce different outcomes in different contexts.

This realist evaluation is the first of its kind to identify theories about how, why and in what conditions nursing staff perceptions vary regarding the CRM of patients' vital signs. Theories regarding nursing staff engagement with remote monitoring are numerous, varied and contradictory. The theories elicited in this initial phase will be refined during interviews with the nursing staff involved with the RCT.

## AUTHOR CONTRIBUTIONS

Candice Downey and Rebecca Randell were involved in the conception of the work and designed the study. Rebecca Randell provided methodological expertise. Candice Downey undertook the data collection and performed the analysis and interpretation. Candice Downey and Rebecca Randell drafted the article. All authors were involved in critical revision of the article and have given final approval of the version to be submitted.

## CONFLICTS OF INTEREST

The authors declare no conflicts of interest.

## ETHICS STATEMENT

Ethical approval for the study was obtained from the Yorkshire & The Humber—Leeds West Research Ethics Committee, REC reference 17/YH/018 on October 13, 2017, and all participants gave informed written consent to enter the study. Participants have consented to the publication of anonymized quotes.

## Supporting information

Supporting information.

Supporting information.

## Data Availability

The datasets used during the current study are available from the corresponding author on reasonable request.
