# Supplementary material for: Nursing staff perspectives of continuous remote vital signs monitoring on surgical wards: Theory elicitation for a realist evaluation
Source: J Eval Clin Pract. 2022 Apr 3;28(3):394–403. doi: 10.1111/jep.13678 (PMC9325470; doi:10.1111/jep.13678)
Supplement: Supplementary file 1 — Supporting information. [file JEP-28-394-s002.docx]

## Supplementary material

### Literature review methodology

A review of the literature was undertaken to identify stakeholders’ ideas about how and in what contexts the optimal use of a new technology is achieved. This represented the theory elicitation stage of a realist review; in a full realist review, published evidence is used to test and refine stakeholders’ theories[7], whereas the purpose of this stage of the work was solely to identify potential theories to be refined in the next stage of the study.

The literature review included grey literature such as editorials, websites of healthcare providers, patient portals and patient information websites, where stakeholders’ ideas are most likely to be found. In addition, the introduction and discussion sections of systematic reviews and primary research studies were examined as these have also been found to contain such theories[6].

##### *Search* *strategy*

MEDLINE®, MEDLINE® In-Process, EMBASE, CINAHL and The Cochrane Library were searched for articles published from the dates of inception of the databases (the earliest being 1947) to October 2017.

The search strategy included a combination of keywords and subject headings related to vital signs (Vital signs OR Vitals OR Heart rate OR Pulse OR Blood pressure OR Respiratory rate OR Temperature OR Oxygen saturation OR Electrocardiograph* OR ECG OR EKG) and monitoring (Observation* OR Monitoring OR Monitor* OR Telemetry OR Oximetry) in combination with keywords Continuous AND Intermittent. The websites of professional journals (*Nursing Times*, *Health Service Journal*) were searched. Finally, a search was run on Google (Google^TM^, Mountain View, CA, USA).  To ensure literature saturation, citations and reference lists of selected studies were reviewed to identify any missed papers.

##### *Selection of studies*

All retrieved abstracts, studies and citations were collected, stored on an EndNote reference management database (Clarivate Analytics, London, U.K.), and reviewed. Publications were selected using a staged review of titles and abstracts, followed by full text review. Selection decisions were recorded in a Microsoft Excel (2007) document. The selection and appraisal of identified papers was based on relevance to the review question, as is the case in the theory elicitation phase of a realist review [6]. Papers were included if they contained theories about staff perceptions regarding continuous remote monitoring of patients’ vital signs. These included empirical studies, theoretical literature, review articles and grey literature. Quality appraisal of the selected papers was not undertaken as the purpose was solely to identify potential theories to be refined in the staff interviews, rather than evaluate the truth of the theories at this stage.

#### Data extraction and synthesis

Theories were extracted from the selected studies by CD, and recorded in a working document with links to the original sources. Similar theories were grouped together and refined as the review progressed. Conflicting theories were also included for exploration in Phase 2 of the realist evaluation, with care being taken to note the context in which these contradictory ideas were founded.
