# Supplementary material for: Nursing staff perspectives of continuous remote vital signs monitoring on surgical wards: Theory elicitation for a realist evaluation
Source: J Eval Clin Pract. 2022 Apr 3;28(3):394–403. doi: 10.1111/jep.13678 (PMC9325470; doi:10.1111/jep.13678)
Supplement: Supplementary file 2 — Supporting information. [file JEP-28-394-s001.docx]

## Supplementary material

**Topic guide for Focus Group 14^th^ September 2017**

**Documentation of attendees** (anonymised)

**Introduction**

- Thanks for helping
- Introductions:

“You have all been involved in a study of a device in the last year.”

- Introduce the aim of the focus group and time schedule:

“The aim of this discussion is to find out about how you found being in the study, and how we can make things better for patients in future studies.”

- Emphasise confidentiality and anonymisation
- There are no right or wrong answers:

“This is simply about your experiences.”

- Introduce dictaphone
- Invite questions

**Questions**

Start from the beginning:

- Why did you agree to be involved in the trial?

- Altruism: some people are willing to take part in research that will only benefit future patients, but not the person themselves.

- Hope of personal benefit?

- What were your expectations of being in the trial?
- Who influenced your decision to be involved?

- Just the patient

- Relatives

- Clinician

- Research team?

- Did you feel that you understood what the trial was about? Did you know it was research?
- The device is very new and hasn’t been proven. Would you have agreed to a trial of a new drug or a new surgery?
- Did you know that other people in the study wouldn’t be given the patch?
- Would you have agreed to be in this group?
- How did you feel when you were told that you had been put into the group wearing the patch?
- In one of the studies, patients can be given a dummy device, which doesn’t work, so that we can compare the real device. How would you feel if we couldn’t tell you which treatment you were getting?
- Did you enjoy being in the study?
- What were the good things about being in the study?
- Extra attention
- Did you feel you benefited physically?
- Did you feel you benefited psychologically?
- What were the bad things about being in the study? Did anything disappoint you?
- Time for consent?
- Too many visits?
- Too few visits?
- Would you change anything about the study in the future?
- Would you have liked to have been involved in the design of the trial?
- What influenced your decision to come here?
- Money
- Social interaction
- Wanted to share your views
- Would you want to know the results of the trial once it was finished?
- How do you think the results should be used?

**Conclusion**

- Signpost the end of the focus group
- Invite further questions
- Thanks for taking part
- Explain how information will be used
- Vouchers
